# Concomitant administration of sGC stimulators with common classes of anti-hypertensive agents results in increased efficacy in spontaneously hypertensive rats

**DOI:** 10.1186/2050-6511-16-S1-A54

**Published:** 2015-09-02

**Authors:** Peter Germano, Jenny Tobin, Robert Jefferson, Courtney Shea, Adaline Smith, G-Yoon Jamie Im, James Sheppeck, James Wakefield, Kristie Sykes, Maria Ribadeneira, Samuel Rivers, Jaime Masferrer

**Affiliations:** 1 Ironwood Pharmaceuticals Inc., Cambridge, MA, USA

## Background

Soluble guanylate cyclase (sGC) stimulators demonstrate smooth muscle relaxation and vasodilation via the nitric oxide (NO)-sGC-cyclic guanosine monophosphate (cGMP) pathway. A novel class of sGC stimulators, the pyrazole-pyrimidines, was synthesized with the objective of creating a potent, once-a-day (QD) oral treatment for cardiovascular diseases. Several compounds from this class were identified as potent stimulators of sGC *in vitro* (EC_50_ = 40-287 nM). These compounds were evaluated in pharmacokinetic (PK) and blood pressure pharmacodynamics (PD) in vivo rat and dog models and were shown to exhibit sustained compound exposure (T_half_ = >7 hours in preclinical species) after oral dosing, predicting QD dosing in humans. Further, they significantly decreased mean arterial blood pressure (MAP (≥ 10mmHg) after oral dosing. The potential for sGC stimulators to work in combination with reference antihypertensive therapies was assessed in an in vivo PD assay in a spontaneous hypertensive rat (SHR) model. Doses of losartan, atenolol, amlodipine, and our sGC stimulators that induced an effect (< 30mmHg) on MAP were chosen. IWP-121, a representative sGC stimulator, was shown to provide additional MAP lowering effects when combined with losartan, atenolol, or amlodipine, resulting in an increase in overall blood pressure effects between 5-50%. By linking compound concentration to blood pressure change for each compound alone and in combination, we were able to assess the PK/PD relationships for the individual and combined effects.

## Conclusion

sGC stimulators from the pyrazole-pyrimidine class demonstrated potent effects in lowering blood pressure in rats and dogs with a PK profile consistent with predicted once a day dosing in humans. Furthermore, sGC stimulator(IWP-121) enhanced the blood pressure lowering effects of standard anti-hypertensive agents in the rat and may provide opportunities for treating patients with resistant hypertension.

